# The Effect of a Mediterranean and Western Diet on Cognitive Performance in Middle‐Aged Rats

**DOI:** 10.1002/alz.090762

**Published:** 2025-01-03

**Authors:** Rebecca J Solch‐Ottaiano, Madison Prats, Elizabeth Engler‐Chiurazzi, Gregory J Bix, Demetrius M Maraganore

**Affiliations:** ^1^ Tulane University, New Orleans, LA USA

## Abstract

**Background:**

Clinical studies indicate that mid‐life dietary patterns are a risk factor for cognitive decline. Adherence to a Mediterranean diet (MeDi) may promote healthy brain aging in contrast to a Western diet (WD), yet these diets have not been examined in pre‐clinical models. We hypothesized that consumption of the MeDi would have better cognitive performance compared to the Western diet in middle‐aged rats.

**Method:**

A subset of animals that provided diet‐specific fecal samples were used in this pilot study. Four‐month‐old male Fischer 344 rats were randomly assigned to a MeDi (n = 13) or WD (n = 7) for approximately one year. Animals then underwent neurobehavioral assessments including the Y‐maze, Morris water maze, and water radial arm maze to assess aspects of short‐ and long‐term memory. Data were analyzed via Student’s T‐test or Two‐Way Repeated Measures ANOVA where appropriate.

**Result:**

For the Y‐maze, there was no difference in percent correct alternations between diet groups (p = 0.24), but the MeDi group had more total alternations compared to the WD group (p = 0.04). For the Morris Water Maze, the MeDi group had a decreased latency compared to the WD group (p = 0.03). Additionally, there was a Day x Diet Effect where the MeDi group had a decreased latency on days 3‐5 (p ≤ 0.01) compared to the WD group. There was no difference between diet groups for platform probe testing (p = 0.80). For the water radial arm maze, there was no difference in total (p = 0.56), working memory correct (p = 0.73), or working memory incorrect (p = 0.33) errors between diet groups. However, the WD group committed more reference memory errors compared to the MeDi group (p = 0.01).

**Conclusion:**

Middle‐aged animals consuming a MeDi demonstrated better aspects of learning and memory compared to animals consuming a WD. This data helps support clinical data suggesting that mid‐life dietary patterns impact cognitive function.

